# Supplementary material for: Transcription Factor SmSPL2 Inhibits the Accumulation of Salvianolic Acid B and Influences Root Architecture
Source: Int J Mol Sci. 2022 Nov 4;23(21):13549. doi: 10.3390/ijms232113549 (PMC9655873; doi:10.3390/ijms232113549)
Supplement: Supplementary file 1 [file ijms-23-13549-s001.zip › ijms-1983150-supplementary.pdf]

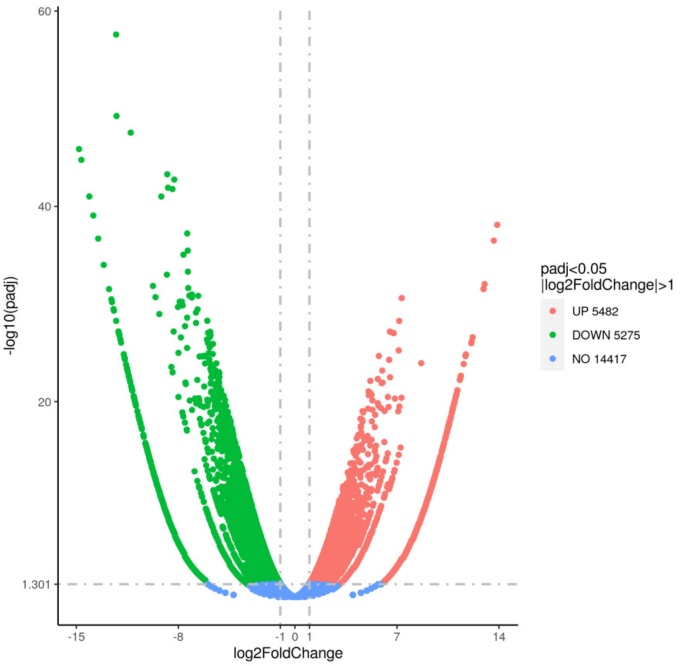

Figure S1. Volcano plot. RNA-seq analysis was performed with *rSmSPL2*-OE2 grown for two-months. A total of 25174 genes were detected of which 5482 were significantly up-regulated and 5272 were significantly down-regulated.

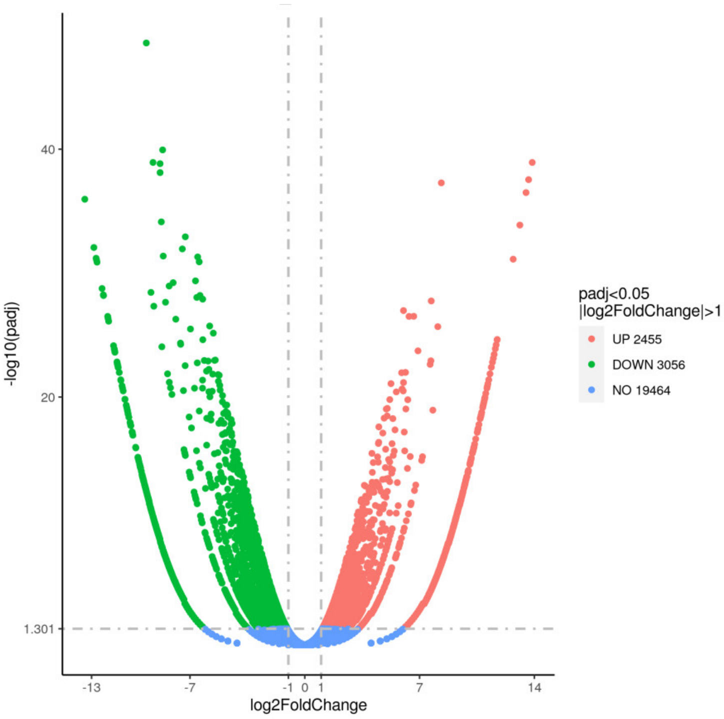

Figure S2. Volcano plot. RNA-seq analysis was performed with *rSmSPL2*-OE7 grown for two-months. A total of 24975 genes were detected of which 2455 were significantly up-regulated and 3056 were significantly down-regulated.

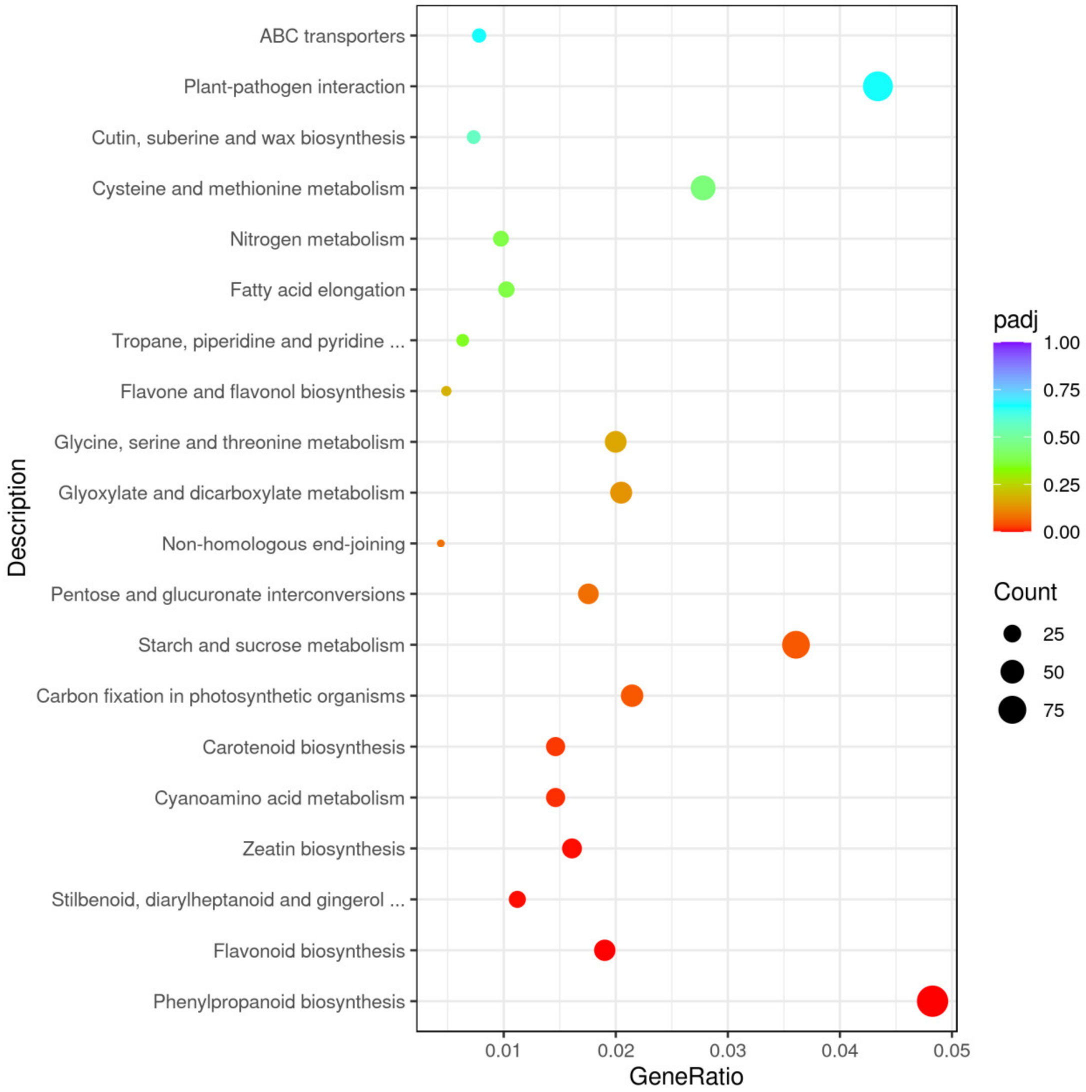

Figure S3. KEGG pathway enrichment analysis of differentially expressed genes (DEGs) in *rSmSPL2*-OE2.

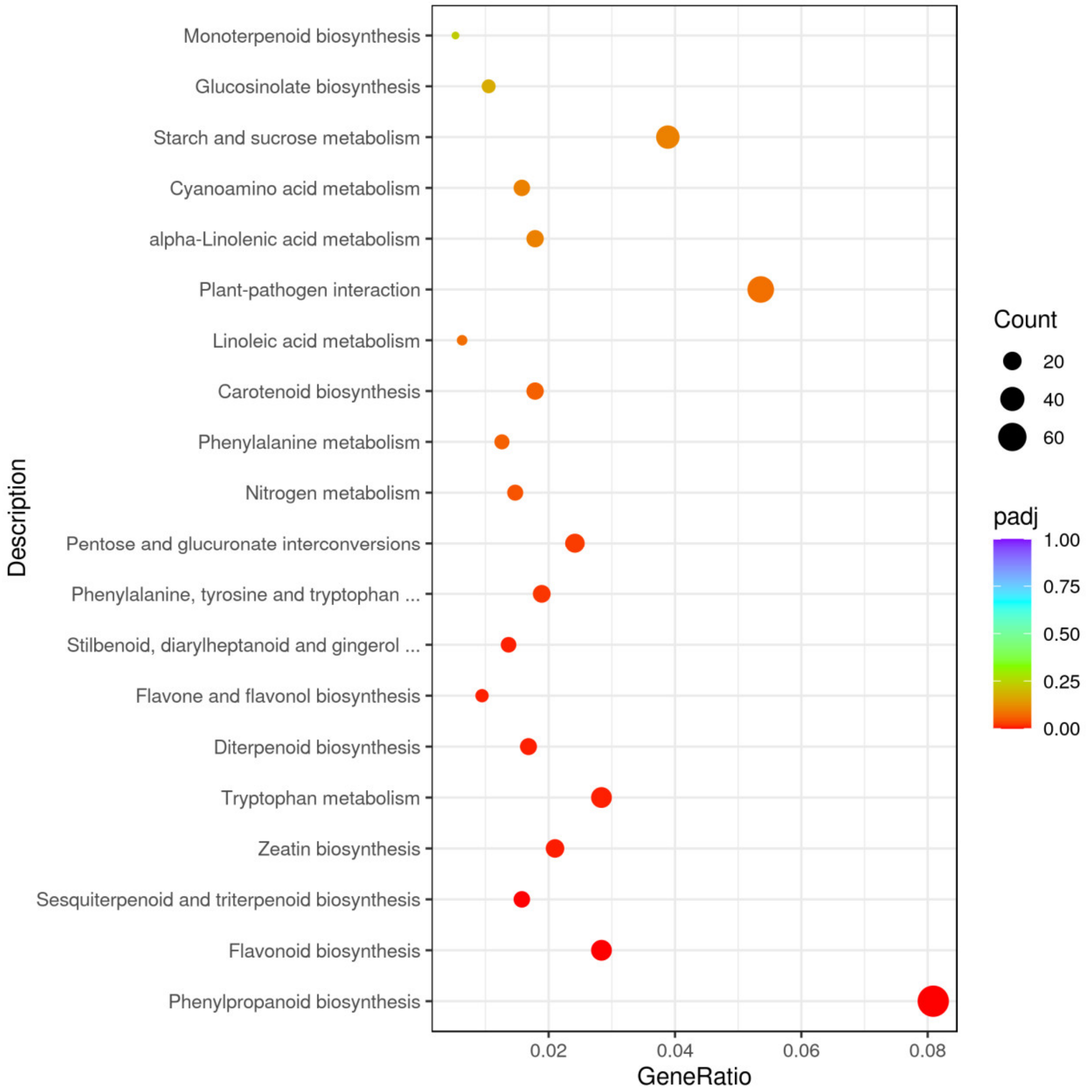

Figure S4. KEGG pathway enrichment analysis of differentially expressed genes (DEGs) in *rSmSPL2*-OE7.

Table S1. List of primers used in the study.

	Primers used for vector construction
SmSPL2-F	ATGAGCCACTTTATGGAGAT
rSmSPL2-A-F	GCTCCGAACGCCCCAATTTTTTGCCAGGTCGAGGGCTGCAACGTT
rSmSPL2-A-R	CTCGACCTGGCAAAAAATTGGGGCGTTCGGAGCAGATGATTTAGT
rSmSPL2-B-F	TATCGTCGCGCCCTATCACTCCTATCT AGCAATTCCTGGGATTCG
rSmSPL2-B-R	ATTGCTAGATAGGAGTGATAGGGCGCGACGATATTCTGGTGCTGC
SmSPL2-R	TCAGTTCAATATATTGGAATAGAA
RT-*SmSPL2*-F	CATTCAAAGTGCCCCAAGGT
RT-*SmSPL2*-R	ATCAGGTGGGGATTGTTCA
RT-*SmUbiquitin*-F	ACCCTCACGGGGAAGACCATC
RT-*SmUbiquitin*-R	ACCACGGAGACGGAGGACAAG
Pro*SmSPL2*-F	CCTCCTTTTCTTTACTGGGTAA
Pro*SmSPL2*-R	AAAAGGAGTAAAGAAGTAAATGCAC
RT -*TAT1*- F	CGAGCAGGGATGGGAGGTTG

RT -*TAT1*- R	GCCTCTTGGCTGTCTCAGCA
RT -*PAL1*- F	GATAGCGGAGTGCAGGTCGTAC
RT -*PAL1*- F	CGAACTAGCAGATTGGCAGAGG
RT -*C4H1*- F	CCAGGAGTCCAAATAACAGAGCCG
RT -*C4H1*- R	GCCACCAAGCGTTCACCAAGAT
RT -*4CL1*- F	TCACCCATGCCGGATTCGAG
RT -*4CL1*- R	AGATCGCGCCGATGAAGGAG
RT -*RAS1*- F	CCAAAGTCAATTATGCCAAGGG
RT -*RAS1*- R	GTCGGATAGGTGGTGCTCGT
RT -*CYP98A14*- F	CCAATCCTACGGCCCGATCC
RT -*CYP98A14*- R	GCCGTCTTCTGCTGAGCTTGA
SmSPL2- pGADT7 -F	TCCCCCGGGATGAGCCACTTTATGGAGAT
SmSPL2- pGADT7 -R	CGAGCTCTCAGTTCAATATATTGGAATAGAA
SmSPL2- 62sk-F	TCCCCCGGGATGAGCCACTTTATGGAGAT
SmSPL2- 62sk-R	GGGGTACCTCAGTTCAATATATTGGAATAGAA

Table S2. List of Gene ID used in the study.

Gene ID*	
EVM0027081	*TAT1*
EVM0018793	*TAT2*
EVM0025031	*TAT3*
EVM0015799	*HPPR1*
EVM0011757	*HPPR2*
EVM0015799	*HPPR3*
EVM0011907	*PAL1*
EVM0024933	*PAL2*
EVM0011707	*PAL3*
EVM0008504	*C4H1*
EVM0027474	*4CL1*
EVM0010662	*4CL2*
EVM0003044	*4CL3*
EVM0012984	*4CL4*
EVM0010773	*4CL5*
EVM0021739	*4CL6*
EVM0025441	*4CL7*
EVM0016577	*RAS1*
EVM0004296	*RAS2*
EVM0012270	*RAS3*
EVM0020785	*CYP98A14*
EVM0008588	*IPT1*
EVM0027273	*IPT2*
EVM0025311	*CYP735A1*

EVM0019778	*CYP735A2*
EVM0007859	*LOG1*
EVM0012385	*LOG2*
EVM0022301	*LOG3*
EVM0003936	*LOG6*
EVM0017980	*AHP1*
EVM0016109	*AHP2*
EVM0011500	*B-RR1*
EVM0015281	*B-RR2*
EVM0008754	*ARR1*
EVM0011715	*ARR2*
EVM0010278	*ARR3*
EVM0007962	*ARR4*
EVM0025386	*TAA1*
EVM0018350	*TAA2*
EVM0013469	*A1M1*
EVM0020376	*AAO1*
EVM0006475	*IAA1*
EVM0010879	*IAA3*
EVM0025242	*IAA9*
EVM0026407	*IAA12*
EVM0010171	*IAA19*
EVM0009395	*IAA21*
EVM0004349	*IAA23*
EVM0009359	*GH3.1*
EVM0004989	*GH3.2*
EVM0001145	*GH3.3*
EVM0007471	*GH3.4*
EVM0024946	*GH3.5*
EVM0026618	*GH3.6*
EVM0018330	*GH3.8*
EVM0017956	*SAUR*
